# Genome-wide meta-analysis for Alzheimer’s disease cerebrospinal fluid biomarkers

**DOI:** 10.1007/s00401-022-02454-z

**Published:** 2022-09-06

**Authors:** Iris E. Jansen, Sven J. van der Lee, Duber Gomez-Fonseca, Itziar de Rojas, Maria Carolina Dalmasso, Benjamin Grenier-Boley, Anna Zettergren, Aniket Mishra, Muhammad Ali, Victor Andrade, Céline Bellenguez, Luca Kleineidam, Fahri Küçükali, Yun Ju Sung, Niccolo Tesí, Ellen M. Vromen, Douglas P. Wightman, Daniel Alcolea, Montserrat Alegret, Ignacio Alvarez, Philippe Amouyel, Lavinia Athanasiu, Shahram Bahrami, Henri Bailly, Olivia Belbin, Sverre Bergh, Lars Bertram, Geert Jan Biessels, Kaj Blennow, Rafael Blesa, Mercè Boada, Anne Boland, Katharina Buerger, Ángel Carracedo, Laura Cervera-Carles, Geneviève Chene, Jurgen A. H. R. Claassen, Stephanie Debette, Jean-Francois Deleuze, Peter Paul de Deyn, Janine Diehl-Schmid, Srdjan Djurovic, Oriol Dols-Icardo, Carole Dufouil, Emmanuelle Duron, Emrah Düzel, EADB consortium, Tormod Fladby, Juan Fortea, Lutz Frölich, Pablo García-González, Maria Garcia-Martinez, Ina Giegling, Oliver Goldhardt, Johan Gobom, Timo Grimmer, Annakaisa Haapasalo, Harald Hampel, Olivier Hanon, Lucrezia Hausner, Stefanie Heilmann-Heimbach, Seppo Helisalmi, Michael T. Heneka, Isabel Hernández, Sanna-Kaisa Herukka, Henne Holstege, Jonas Jarholm, Silke Kern, Anne-Brita Knapskog, Anne M. Koivisto, Johannes Kornhuber, Teemu Kuulasmaa, Carmen Lage, Christoph Laske, Ville Leinonen, Piotr Lewczuk, Alberto Lleó, Adolfo López de Munain, Sara Lopez-Garcia, Wolfgang Maier, Marta Marquié, Merel O. Mol, Laura Montrreal, Fermin Moreno, Sonia Moreno-Grau, Gael Nicolas, Markus M. Nöthen, Adelina Orellana, Lene Pålhaugen, Janne M. Papma, Florence Pasquier, Robert Perneczky, Oliver Peters, Yolande A. L. Pijnenburg, Julius Popp, Danielle Posthuma, Ana Pozueta, Josef Priller, Raquel Puerta, Inés Quintela, Inez Ramakers, Eloy Rodriguez-Rodriguez, Dan Rujescu, Ingvild Saltvedt, Pascual Sanchez-Juan, Philip Scheltens, Norbert Scherbaum, Matthias Schmid, Anja Schneider, Geir Selbæk, Per Selnes, Alexey Shadrin, Ingmar Skoog, Hilkka Soininen, Lluís Tárraga, Stefan Teipel, The GR@ACE study group, Betty Tijms, Magda Tsolaki, Christine Van Broeckhoven, Jasper Van Dongen, John C. van Swieten, Rik Vandenberghe, Jean-Sébastien Vidal, Pieter J. Visser, Jonathan Vogelgsang, Margda Waern, Michael Wagner, Jens Wiltfang, Mandy M. J. Wittens, Henrik Zetterberg, Miren Zulaica, Cornelia M. van Duijn, Maria Bjerke, Sebastiaan Engelborghs, Frank Jessen, Charlotte E. Teunissen, Pau Pastor, Mikko Hiltunen, Martin Ingelsson, Ole A. Andreassen, Jordi Clarimón, Kristel Sleegers, Agustín Ruiz, Alfredo Ramirez, Carlos Cruchaga, Jean-Charles Lambert, Wiesje van der Flier

**Affiliations:** 1 Alzheimer Center Amsterdam, Neurology, Vrije Universiteit Amsterdam, Amsterdam UMC Location VUmc, Amsterdam, The Netherlands; 2 Amsterdam Neuroscience, Neurodegeneration, Amsterdam, The Netherlands; 3 Department of Complex Trait Genetics, Center for Neurogenomics and Cognitive Research, Amsterdam Neuroscience, VU Amsterdam, Amsterdam, The Netherlands; 4 Section Genomics of Neurodegenerative Diseases and Aging, Human Genetics, Vrije Universiteit Amsterdam, Amsterdam UMC location VUmc, Amsterdam, The Netherlands; 5 Department of Psychiatry, Washington University School of Medicine, St Louis, MO USA; 6 NeuroGenomics and Informatics, Washington University School of Medicine, St Louis, MO USA; 7 Hope Center for Neurological Disorders, Washington University School of Medicine, St Louis, MO USA; 8 Research Center and Memory Clinic, Ace Alzheimer Center Barcelona, Universitat Internacional de Catalunya, Barcelona, Spain; 9 CIBERNED, Network Center for Biomedical Research in Neurodegenerative Diseases, National Institute of Health Carlos III, Madrid, Spain; 10 Division of Neurogenetics and Molecular Psychiatry, Department of Psychiatry and Psychotherapy, Faculty of Medicine and University Hospital Cologne, University of Cologne, Cologne, Germany; 11 Neurosciences and Complex Systems Unit (ENyS), CONICET, Hospital El Cruce, National University A. Jauretche (UNAJ), Florencio Varela, Argentina; 12 Univ. Lille, Inserm, CHU Lille, Institut Pasteur de Lille, U1167 - RID-AGE / Labex DISTALZ - Facteurs de risque et déterminants moléculaires des maladies liées au vieillissement, F-59000 Lille, France; 13 Neuropsychiatric Epidemiology Unit, Department of Psychiatry and Neurochemistry, Institute of Neuroscience and Physiology, The Sahlgrenska Academy, Centre for Ageing and Health (AGECAP) at the University of Gothenburg, Gothenburg, Sweden; 14 University of Bordeaux, Inserm, Bordeaux Population Health Research Center, Team VINTAGE, UMR 1219, 33000 Bordeaux, France; 15 Department of Neurodegenerative Diseases and Geriatric Psychiatry, University Hospital Bonn, Medical Faculty, Bonn, Germany; 16 German Center for Neurodegenerative Diseases (DZNE), Bonn, Germany; 17 Complex Genetics of Alzheimer’s Disease Group, VIB Center for Molecular Neurology, VIB, Antwerp, Belgium; 18 Department of Biomedical Sciences, University of Antwerp, Antwerp, Belgium; 19 Sant Pau Memory Unit, Department of Neurology, Institut d’Investigacions Biomèdiques Sant Pau - Hospital de Sant Pau, Universitat Autònoma de Barcelona, Barcelona, Spain; 20 Memory Disorders Unit, Department of Neurology, Hospital Universitari Mutua de Terrassa, Terrassa, Spain; 21 Fundació per a la Recerca Biomèdica i Social Mútua de Terrassa, Terrassa, Spain; 22 NORMENT Centre, Institute of Clinical Medicine, University of Oslo and Division of Mental Health, Oslo, Norway; 23 Université Paris Cité, EA4468, Maladie d’Alzheimer, F-75013 Paris, France; 24 The Research-Centre for Age-Related Functional Decline and Disease, Innlandet Hospital Trust, Brumunddal, Norway; 25 Norwegian National Centre for Ageing and Health, Vestfold Hospital Trust, Tønsberg, Norway; 26 Lübeck Interdisciplinary Platform for Genome Analytics, University of Lübeck, Lübeck, Germany; 27 Department of Neurology, UMC Utrecht Brain Center, Utrecht, The Netherlands; 28 Department of Psychiatry and Neurochemistry, Institute of Neuroscience and Physiology, The Sahlgrenska Academy at the University of Gothenburg, Mölndal, Sweden; 29 Clinical Neurochemistry Laboratory, Sahlgrenska University Hospital, Mölndal, Sweden; 30 Université Paris-Saclay, CEA, Centre National de Recherche en Génomique Humaine, 91057 Evry, France; 31 German Center for Neurodegenerative Diseases (DZNE, Munich), Munich, Germany; 32 Institute for Stroke and Dementia Research (ISD), University Hospital, LMU Munich, Munich, Germany; 33 Grupo de Medicina Xenómica, Centro Nacional de Genotipado (CEGEN-PRB3-ISCIII), Universidade de Santiago de Compostela, Santiago de Compostela, Spain; 34 Fundación Pública Galega de Medicina Xenómica-CIBERER-IDIS, Santiago de Compostela, Spain; 35 Department of Neurology, CHU de Bordeaux, 33000 Bordeaux, France; 36 Radboudumc Alzheimer Center, Department of Geriatrics, Radboud University Medical Center, Nijmegen, The Netherlands; 37 Donders Center for Medical Neuroscience, Nijmegen, The Netherlands; 38 Department of Neurology, Boston University School of Medicine, Boston, MA 2115 USA; 39 Department of Neurology and Alzheimer Center Groningen, University Medical Center Groningen, Groningen, The Netherlands; 40 Center for Cognitive Disorders, Department of Psychiatry and Psychotherapy, Klinikum rechts der Isar, Technical University of Munich, School of Medicine, Munich, Germany; 41 kbo-Inn-Salzach-Hospital, Wasserburg am Inn, Germany; 42 Department of Medical Genetics, Oslo University Hospital, Oslo, Norway; 43 Department of Clinical Science, NORMENT Centre, University of Bergen, Bergen, Norway; 44 Pôle de Santé Publique Centre Hospitalier Universitaire (CHU) de Bordeaux, Bordeaux, France; 45 Univerisité Paris-Saclay. Inserm 1178 MOODS, Paris, France; 46 German Center for Neurodegenerative Diseases (DZNE), Magdeburg, Germany; 47 Institute of Cognitive Neurology and Dementia Research (IKND), Otto-von-Guericke University, Magdeburg, Germany; 48 Institute of Clinical Medicine, University of Oslo, Oslo, Norway; 49 Department of Neurology, Akershus University Hospital, Lorenskog, Norway; 50 Department of Geriatric Psychiatry, Central Institute of Mental Health, Medical Faculty Mannheim, Heidelberg, Germany; 51 Cognitive Impairment Unit, Neurology Service, “Marqués de Valdecilla” University Hospital, Institute for Research “Marques de Valdecilla” (IDIVAL), University of Cantabria, Santander, Spain, and Centro de Investigación Biomédica en Red sobre Enfermedades Neurodegenerativas (CIBERNED), Madrid, Spain; 52 Division of General Psychiatry, Department of Psychiatry and Psychotherapy, Medical University of Vienna, Vienna, Austria; 53 A.I. Virtanen Institute for Molecular Sciences, University of Eastern Finland, Kuopio, Finland; 54 Alzheimer Precision Medicine (APM), Sorbonne University, AP-HP, Pitié-Salpêtrière Hospital, Paris, France; 55 Neurology Business Group, Eisai Inc, 100 Tice Blvd, Woodcliff Lake, NJ 07677 USA; 56 Service gériatrie, Centre Mémoire de Ressources et Recherches Ile de France-Broca, AP-HP, Hôpital Broca, F-75013 Paris, France; 57 Institute of Human Genetics, University of Bonn, School of Medicine and University Hospital Bonn, 53127 Bonn, Germany; 58 Institute of Clinical Medicine, Internal Medicine, University of Eastern Finland, Kuopio, Finland; 59 Department of Neurology, Institute of Clinical Medicine, University of Eastern Finland, Kuopio, Finland; 60 Department of Neurology, Akershus University Hospital, Lorenskog, Norway; 61 Region Västra Götaland, Sahlgrenska University Hospital, Psychiatry, Cognition and Old Age Psychiatry Clinic, Gothenburg, Sweden; 62 Department of Geriatric Medicine, Oslo University Hospital, Oslo, Norway; 63 Department of Neurology, Kuopio University Hospital, Kuopio, Finland; 64 Department of Neurology, Helsinki University Hospital, Helsinki, Finland; 65 Department of Psychiatry and Psychotherapy, Universitätsklinikum Erlangen, and Friedrich-Alexander Universität Erlangen-Nürnberg, Erlangen, Germany; 66 Bioinformatics Center, Institute of Biomedicine, University of Eastern Finland, Kuopio, Finland; 67 Atlantic Fellow at the Global Brain Health Institute (GBHI) -, University of California, San Francisco, USA; 68 German Center for Neurodegenerative Diseases (DZNE), Tübingen, Germany; 69 Section for Dementia Research, Hertie Institute for Clinical Brain Research and Department of Psychiatry and Psychotherapy, University of Tübingen, Tübingen, Germany; 70 Institute of Clinical Medicine, Neurosurgery, University of Eastern Finland, Kuopio, Finland; 71 Department of Neurosurgery, Kuopio University Hospital, Kuopio, Finland; 72 Department of Neurodegeneration Diagnostics, Medical University of Białystok, Białystok, Poland; 73 Hospital Universitario Donostia-OSAKIDETZA, Donostia, Spain; 74 Instituto Biodonostia, San Sebastián, Spain; 75 University of The Basque Country, San Sebastian, Spain; 76 Department of Epidemiology, ErasmusMC, Rotterdam, The Netherlands; 77 Department of Genetics and CNR-MAJ, Normandie Univ, UNIROUEN, Inserm U1245 and CHU Rouen, Rouen, France; 78 Department of Neurology and Alzheimer Center Erasmus MC, Erasmus MC University Medical Center, Rotterdam, The Netherlands; 79 Department of Psychiatry and Psychotherapy, University Hospital, LMU Munich, Munich, Germany; 80 Munich Cluster for Systems Neurology (SyNergy) Munich, Munich, Germany; 81 Ageing Epidemiology Research Unit, School of Public Health, Imperial College London, London, UK; 82 German Center for Neurodegenerative Diseases (DZNE), Berlin, Germany; 83 Department of Geriatric Psychiatry, University Hospital of Psychiatry Zürich and University of Zürich, Zurich, Switzerland; 84 Old Age Psychiatry, Department of Psychiatry, University Hospital of Lausanne, Lausanne, Switzerland; 85 Department of Psychiatry and Psychotherapy, Charité, Charitéplatz 1, 10117 Berlin, Germany; 86 Department of Psychiatry and Psychotherapy, Klinikum rechts der isar, Technical University Munich, 81675 Munich, Germany; 87 Department of Psychiatry and Neuropsychologie, Alzheimer Center Limburg, Maastricht University, Maastricht, The Netherlands; 88 Department of Neuromedicine and Movement Science, Norwegian University of Science and Technology (NTNU), Trondheim, Norway; 89 Department of Geriatrics, St Olav Hospital, University Hospital of Trondheim, Trondheim, Norway; 90 Alzheimer’s Centre Reina Sofia-CIEN Foundation-ISCIII, 28031 Madrid, Spain; 91 Department of Psychiatry and Psychotherapy, Medical Faculty, LVR-Hospital Essen, University of Duisburg-Essen, Essen, Germany; 92 Institute of Medical Biometry, Informatics and Epidemiology, University Hospital of Bonn, Bonn, Germany; 93 German Center for Neurodegenerative Diseases (DZNE), Rostock, Germany; 94 Department of Psychosomatic Medicine, Rostock University Medical Center, Gehlsheimer Str. 20, 18147 Rostock, Germany; 95 1st Department of Neurology, School of Medicine, Faculty of Health Sciences, Aristotle University of Thessaloniki, Thessaloniki, Makedonia Greece; 96 Neurodegenerative Brain Diseases Group, VIB Center for Molecular Neurology, VIB, Antwerp, Belgium; 97 Neurology, University Hospitals Leuven, Leuven, Belgium; 98 Laboratory for Cognitive Neurology, Department of Neurosciences, Leuven Brain Institute, Leuven, Belgium; 99 Alzheimer Center Limburg, School for Mental Health and Neuroscience Maastricht University, Maastricht, The Netherlands; 100 Department of Neurobiology, Care Sciences and Society, Division of Neurogeriatrics Karolinska Institutet, Stockholm, Sweden; 101 Department of Psychiatry and Psychotherapy, University Medical Center Goettingen, Göttingen, Germany; 102 Department of Psychiatry, Harvard Medical School, McLean Hospital, Belmont, MA USA; 103 Region Västra Götaland, Sahlgrenska University Hospital, Psychiatry, Psychosis Clinic, Gothenburg, Sweden; 104 German Center for Neurodegenerative Diseases (DZNE), Göttingen, Germany; 105 Medical Science Department, iBiMED, Aveiro, Portugal; 106 Center for Neurosciences (C4N), Vrije Universiteit Brussel, Brussels, Belgium; 107 Department of Neurodegenerative Disease, UCL Institute of Neurology, London, UK; 108 UK Dementia Research Institute at UCL, London, UK; 109 Hong Kong Center for Neurodegenerative Diseases, Hong Kong, China; 110 Nuffield Department of Population Health, Oxford University, Oxford, UK; 111 Laboratory of Neurochemistry, Universitair Ziekenhuis Brussel, Brussels, Belgium; 112 Department of Neurology, Universitair Ziekenhuis Brussel, Brussels, Belgium; 113 Department of Psychiatry and Psychotherapy, Faculty of Medicine and University Hospital Cologne, University of Cologne, Cologne, Germany; 114 Cluster of Excellence Cellular Stress Responses in Aging-Associated Diseases (CECAD), University of Cologne, Cologne, Germany; 115 Neurochemistry Lab, Department of Clinical Chemistry, Amsterdam Neuroscience, Vrije Universiteit Amsterdam, Amsterdam UMC, Amsterdam, The Netherlands; 116 Unit of Neurodegenerative diseases, Department of Neurology, University Hospital Germans Trias i Pujol and The Germans Trias i Pujol Research Institute (IGTP) Badalona, Barcelona, Spain; 117 Institute of Biomedicine, University of Eastern Finland, Kuopio, Finland; 118 Department of Public Health and Caring Sciences, Molecular Geriatrics, Rudbeck Laboratory, Uppsala University, Uppsala, Sweden; 119 Krembil Brain Institute, University Health Network, Toronto, Ontario Canada; 120 Department of Medicine and Tanz Centre for Research in Neurodegenerative Diseases, University of Toronto, Toronto, Canada; 121 Addiction, Oslo University Hospital, 0407 Oslo, Norway; 122 Department of Psychiatry, Glenn Biggs Institute for Alzheimer’s and Neurodegenerative Diseases, San Antonio, TX USA

**Keywords:** GWAS, Alzheimer’s disease, Cerebrospinal fluid, Amyloid-beta, Tau

## Abstract

Amyloid-beta 42 (Aβ42) and phosphorylated tau (pTau) levels in cerebrospinal fluid (CSF) reflect core features of the pathogenesis of Alzheimer’s disease (AD) more directly than clinical diagnosis. Initiated by the European Alzheimer & Dementia Biobank (EADB), the largest collaborative effort on genetics underlying CSF biomarkers was established, including 31 cohorts with a total of 13,116 individuals (discovery *n* = 8074; replication *n* = 5042 individuals). Besides the *APOE* locus, novel associations with two other well-established AD risk loci were observed; *CR1* was shown a locus for Aβ42 and *BIN1* for pTau. *GMNC* and *C16orf95* were further identified as loci for pTau, of which the latter is novel. Clustering methods exploring the influence of all known AD risk loci on the CSF protein levels, revealed 4 biological categories suggesting multiple Aβ42 and pTau related biological pathways involved in the etiology of AD. In functional follow-up analyses, *GMNC* and *C16orf95* both associated with lateral ventricular volume, implying an overlap in genetic etiology for tau levels and brain ventricular volume.

**Supplementary Information:**

The online version contains supplementary material available at 10.1007/s00401-022-02454-z.

## Introduction

Resolving the genetic background of Alzheimer’s disease (AD) has proven to contribute greatly to our understanding of underlying disease processes, for instance with the discovery of *APP* [25], *PSEN1* [53], and *PSEN2* [52] in family-based studies, leading to the amyloid cascade theory [38]. In addition, genome-wide association studies (GWAS) in AD have convincingly highlighted the importance of microglia [33, 56], a finding previously supported by research from other scientific fields [18, 72, 75], and now also widely accepted as a genetic cause rather than a result of AD pathogenesis. Further exploration of genetic risk factors contributing to AD development and pathogenesis might reveal more biological insights, an important step in the quest for AD treatment that will slow down or even halt disease progression.

GWAS of clinically diagnosed AD patients have been successful, and current efforts largely focus on increasing sample size to improve the statistical power to detect genetic variants [6, 70]. An alternative approach is to study effects of genetic variants on pathophysiological features of AD. The strength of such studies is based on the assumption that more objective measurable biological properties are more strongly associated with the underlying AD pathology than the clinical diagnostic classifications (e.g., misclassifications or symptoms not manifested yet), thereby allowing to detect larger effects by reducing heterogeneity [26]. The use of biomarkers further enables to identify genetic effects specific for certain AD-related biological mechanisms. This is an advantage over the conventional GWAS approach for clinical AD diagnosis, where it generally remains unclear through what causal gene or cellular process a locus is associated to AD.

It is possible to measure levels of amyloid-beta-42 (Aβ42) and (phosphorylated) tau (pTau and Tau) in cerebrospinal fluid (CSF), the two major proteins implicated in the AD pathological process. Aβ42 pathology in the brain is negatively correlated with CSF Aβ42 levels, where a decrease in CSF Aβ42 is indicative of AD [49, 58]. CSF (p)Tau is positively correlated with (p)Tau pathology in the brain, and therefore higher CSF (p)Tau levels are observed in patients with AD. CSF pTau is presumed to reflect AD-type tau-tangles more specifically than total tau [49, 58]. Previous studies on CSF amyloid-beta and (p)Tau have identified genetic risk loci, the most recent one including 3,146 individuals [17]. Some of the 8 discovered loci had not been previously associated with AD, emphasizing the potential of endophenotypes to reveal novel genetic risk factors. Our current study aimed to further define the genetic background of AD by studying the genetic effects on CSF Aβ42 and pTau levels in a total of 13,116 individuals.

## Materials and methods

### Participants

**Fig. 1:**
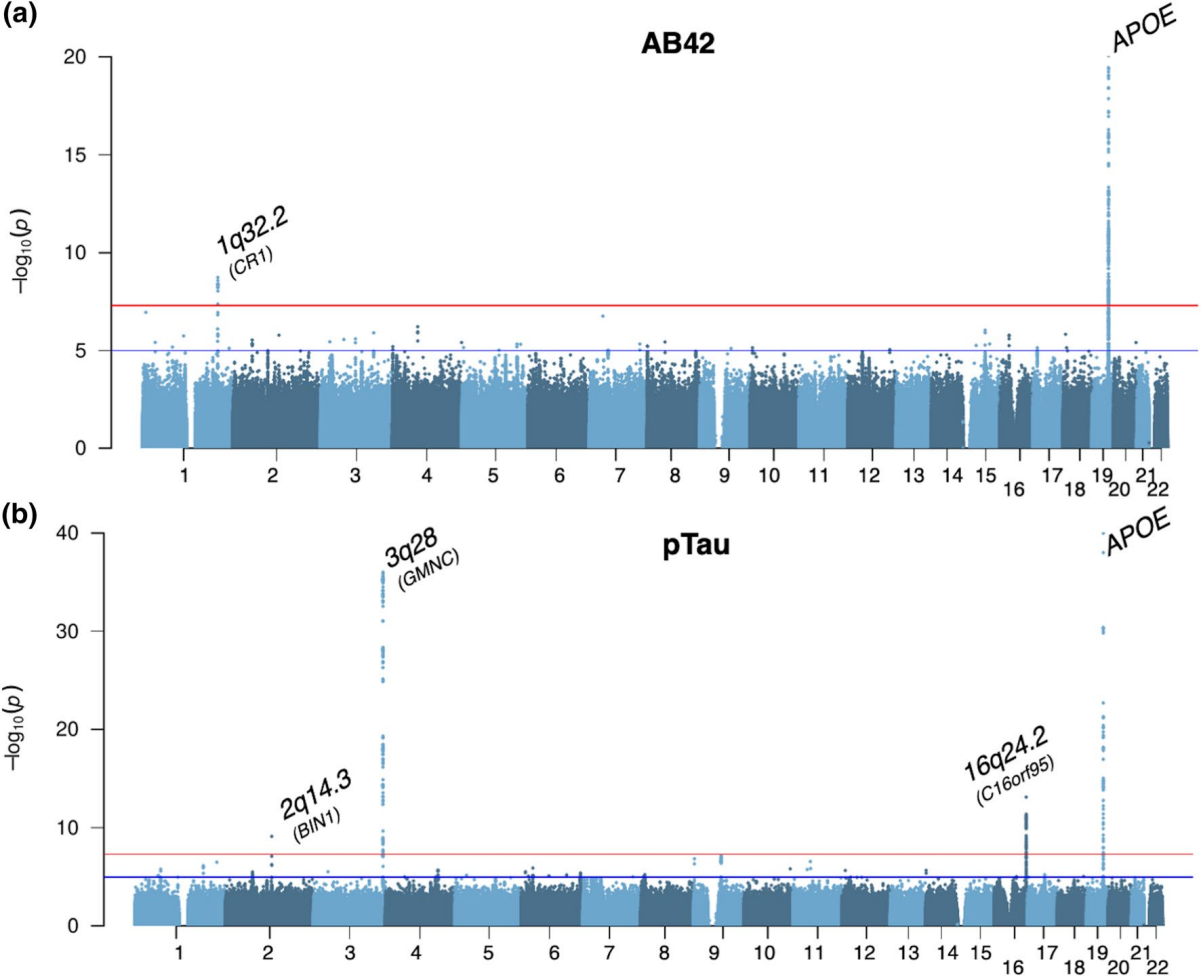
Manhattan plots of the stage 3 GWAS results. **a** Results visualized for CSFAβ42; **b** Results visualized for CSF pTau. The *y*-axes are limited to visualize the non-APOE loci. The lowest *P* values for APOE are 4.07 × 10^–355^ and 3.74 × 10^–94^ for Aβ42 and pTau, respectively

We combined data from 16 European cohorts, encompassing a total of 8074 individuals (Table 1; Online Resource 1—Table 1; Online Resource 2—Fig. 1) with both genotype data and CSF measurements. The majority of these cohorts (82%) are part of the EADB consortium [6], and included the full spectrum of clinical severity potentially leading to AD, from subjective cognitive decline, mild cognitive impairment, to dementia. Written informed consent was obtained from study participants or, for those with substantial cognitive impairment, from a caregiver, legal guardian, or other proxy. Study protocols for all cohorts were reviewed and approved by the appropriate institutional review boards.

**Table 1:** Demographic information on cohorts of stage 1 discovery analysis

Country	Cohort	Age	Gender	Diagnoses				APOE4 carrier	Aβ42		pTau	
		Mean years (sd)	% male	AD (%)	MCI (%)	Control (%)	Other dem (%)	% APOE4 +	Mean levels (sd)	*n*	Mean levels (sd)	*n*
Belgium	DEM	78.3 (7.3)	40	72	27	1	0	48	562.7 (250.8)	587	71.6 (35.4)	585
Finland	ADGEN	70.2 (8.0)	34	89	1	10	0	73	477.1 (183.2)	226	80.4 (34.8)	155
France1	BALTAZAR	77.0 (6.7)	45	43	57	0	0	43	825.7 (358.9)	420	73.0 (34.0)	419
France2	MEMENTO	69.2 (8.9)	47	0	100	0	0	30	1078.6 (406.7)	389	62.6 (29.8)	386
France3	CNRMAJ-Lille	66.0 (8.7)	47	100	0	0	0	56	664.8 (205.1)	127	101.8 (53.4)	125
Germany1	Delcode	71.7 (5.9)	52	13	23	64	0	39	965.3 (338.2)	465	63.2 (35.1)	462
Germany2	KND	67.3 (8.7)	57	18	82	0	0	49	724.0 (347.9)	309	69.4 (36.8)	305
Germany3	TUM	70.2 (9.2)	48	98	1	0	1	58	536.8 (240.0)	151	88.8 (48.7)	152
Germany4	PAGES	73.4 (7.7)	40	70	30	0	0	59	528.2 (245.5)	136	90.8 (60.0)	137
Germany5	UHB	70.3 (7.2)	42	69	30	1	0	68	545.7 (327.8)	111	82.0 (44.2)	111
Netherlands	ADC & Pearl ND	64.1 (8.9)	59	42	10	24	23	50	842.8 (288.9)	2936	65.9 (35.9)	2931
Spain1	ACE	72.7 (8.2)	43	27	59	8	6	36	779.2 (314.3)	609	68.2 (35.5)	609
Spain2	SIGNAL & SPIN	70.6 (8.0)	43	34	45	19	2	39	707.2 (368.3)	394	71.1 (41.9)	370
Spain3	Valdecilla	67.0 (9.0)	39	10	37	45	8	29	887.5 (360.5)	98	56.8 (27.9)	99
Sweden1	Birth cohort & Clin. AD	75.0 (9.4)	45	51	0	49	0	52	533.5 (271.9)	856	68.9 (35.5)	694
Sweden2	Uppsala university	71.0 (6.3)	46	58	37	0	6	64	489.0 (238.1)	260	77.6 (37.2)	259

For most cohorts, one of the two CSF levels is missing for a small number of samples. The demographics for age, gender diagnoses and APOE4 carriers status are then displayed for the largest group of samples with at least one CSF measurement. AB42 levels are corrected according to known drift over time for the Dutch ADC and Pearl ND cohorts. All, except the Swedisch Birth cohort and clinical AD samples, are part of EADB

*Aβ42* amyloid-beta 42, *pTau* phosphorylated tau, *AD* Alzheimer’s disease, *MCI* mild cognitive impairment, *other dem* other dementia, *n* sample size, *DEM* Antwerp prospective dementia cohort

For replication, 15 cohorts totaling 5042 individuals (Online Resource 1—Table 2) were available to attempt replication of the association signals to Aβ42 and pTau, for the variants with *P* value < 1e–5 in the discovery analysis. Data from all cohorts, except one (NorCog from University of Oslo, Norway), were obtained through collaboration with the previous largest GWAS on CSF Aβ42 and pTau, mostly including cohorts originating from the United States [17]. Basic demographics are described in Online Resource 1—Table 2, more detailed cohort information is described elsewhere [17].

### CSF measurements

**Fig. 2:**
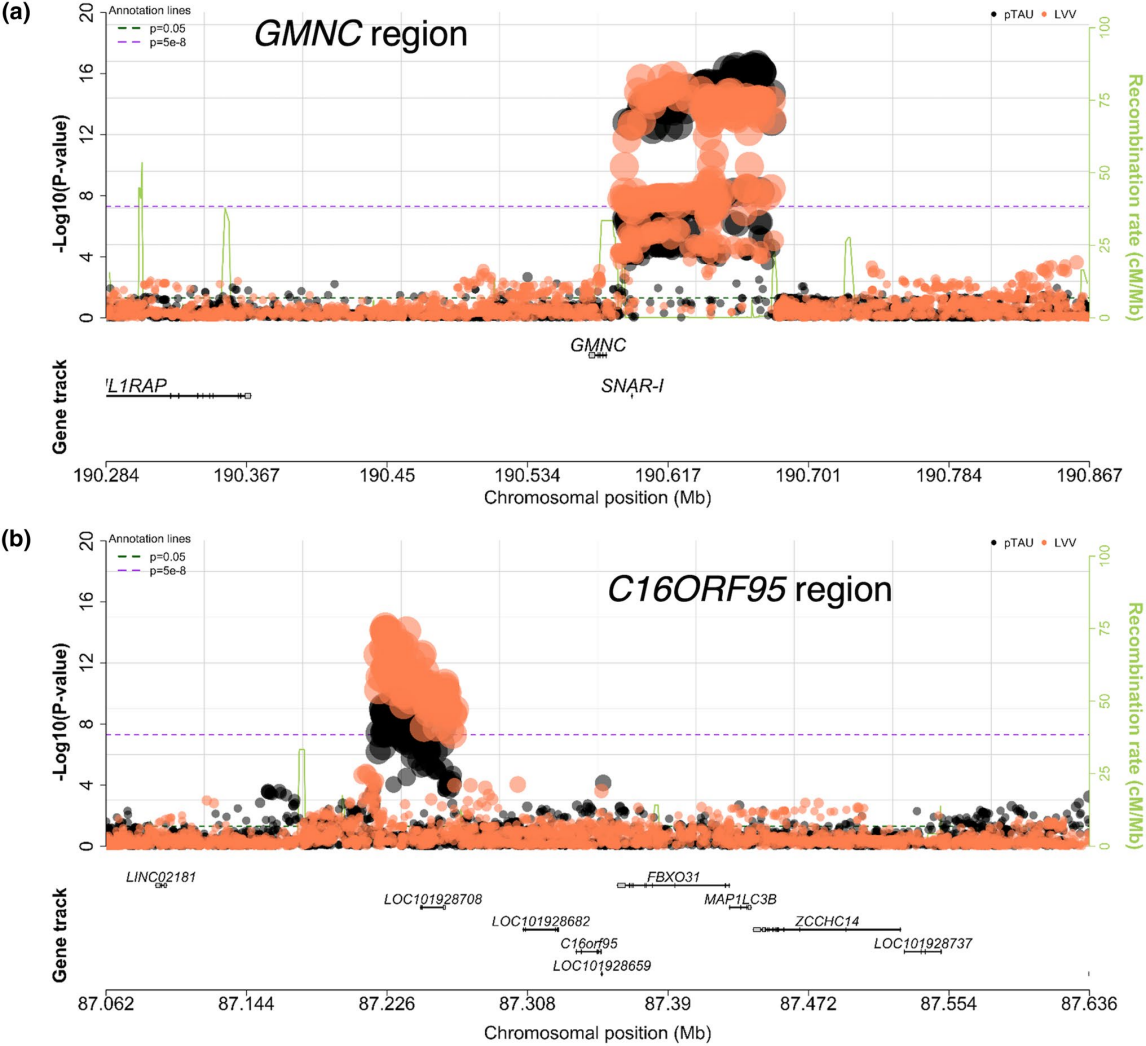
LocusZoom plots showing variant association results for **a** *GMNC* and **b** *C16orf9*5 loci. In black, the pTau association signals of this study; and in orange, the lateral ventricular volume (LVV) association signals observed in other studies

**Fig. 3:**
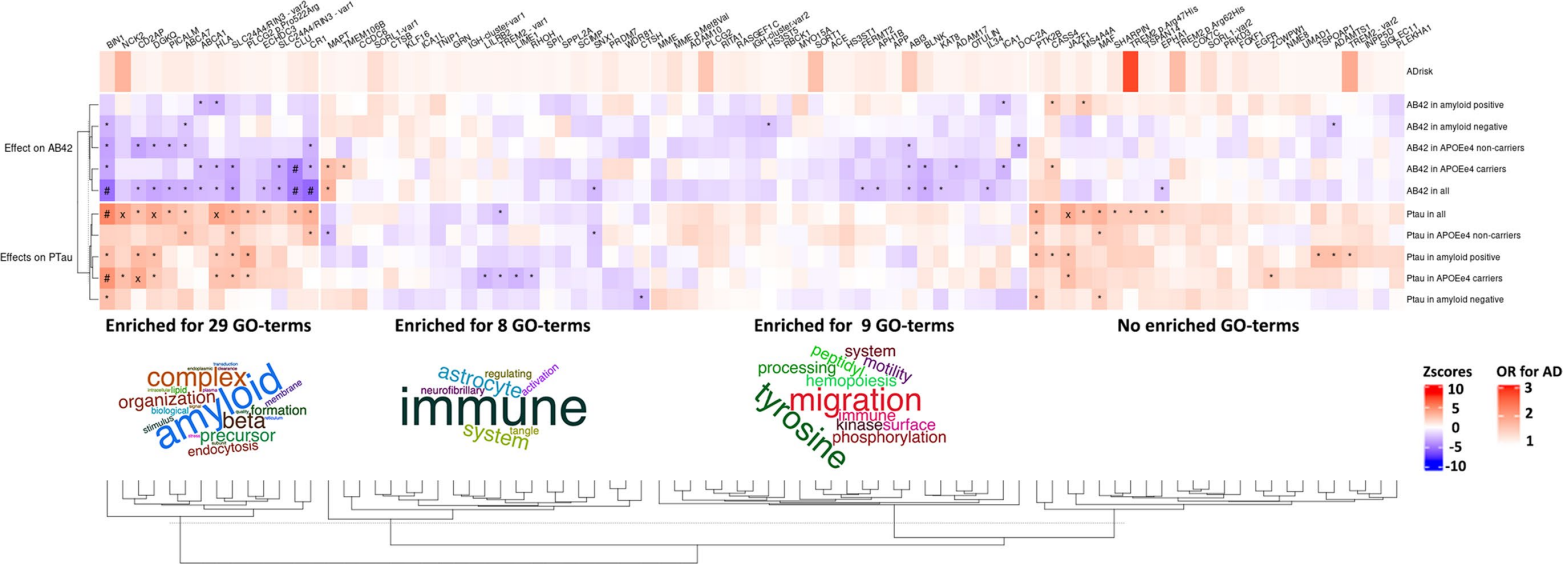
The effects of all AD-associated loci. The names of the loci are named according to their linked gene names in Bellinguez et al. (2021). Hierarchical clustering was performed on the rows and columns using Eucledian distances and the method ‘average’ for clustering Pathway enrichment analyses was performed on the four first clusters. The enrichment analyses are in Online Resource 1—Table 9. The upper bar shows the odds ratio for AD, where alleles for variants with protective effects have been flipped to show AD-risk increasing effects for all variants. The increases in Aβ42 and pTau (positive Zscores) are shown in red and decreases in AB42 and PTau (negative *Z* scores) are shown in blue. * = *P* value < 0.05, *X* = 0.05 < *P* value < 0.001, # = 0.001 < *P* value < 5 × 10^–8^, $ = *P* value < 5 × 10^–8^

Due to the multi-center approach, CSF protein levels were measured with various CSF protein assays (Online Resource 1—Table 2). Aβ42 was measured with ELISA, Lumipulse or V-PLEX, and pTau with ELISA or Lumipulse. For details on specific lab procedures, see the original studies [2–4, 17, 21, 23, 27, 34, 36, 42, 47, 50, 57, 62, 78]. Protein levels were log10 transformed and normalized within cohorts and CSF assay type (if multiple assays were used within a single cohort) to approximate a normal distribution to correct for the application of various CSF assays across different studies. Then, the normalized protein levels were used as continuous phenotypes in the association analyses. The distribution of raw and normalized CSF protein levels are displayed in Online Resource 2—Figs. 2–13, for the cohorts for which we have individual-level data available (indicated in Online Resource 1—Table 1; column D). For the stratified analyses, two subgroups of individuals, amyloid normal and amyloid abnormal, were defined based on their Aβ42 status. Individuals with an untransformed Aβ42 level below a threshold were assigned to the abnormal amyloid level group. The thresholds were defined by the individual research groups as it depends on technical circumstances, and are displayed in Online Resource 1—Table 2.

### Genotyping, quality control and imputation

The genetic data for the EADB cohorts have been processed in a homogeneous approach (Online Resource 1—Table 1), in which the Illumina Infinium Global Screening Array (GSA, GSAsharedCUSTOM_24 + v1.0) was predominantly used for data generation. Additional arrays included the Axiom 815 K Spanish biobank array (Thermo Fisher) for ACE (Barcelona, Spain) and Valdecilla (Santander, Spain) cohorts, and the Illumina Neurochip array (Gothenburg, Sweden). Standard quality control (QC) procedures were performed to exclude individuals and variants with low quality, in general followed by imputation with the Trans-Omics for Precision Medicine (TOPMed) reference panel [13, 59]. For the EADB cohorts for which GSA genotype level data were available, the details on QC steps and imputation with the TOPMed reference panel were previously described [6].

For the Spanish ACE and Valdecilla cohorts, QC procedures are described in another study [16], followed by imputation with the TOPMed reference panel. For the Gothenburg H70 Birth Cohort studies and clinical AD samples from Sweden, the QC and imputation procedures were described elsewhere [48]. Post‐imputation QC only included variants with a high imputation quality (RSQ [imputation quality] > 0.8). The UCSC LiftOver program (https://genome-store.ucsc.edu/) and Plink v2.0 (www.cog-genomics.org/plink/2.0/) [10] were used to lift the GRCh37 genomic positions to GRCh38, the genomic build for all other datasets. All genotypes were hard called using the default Plink v2.0 (http://www.cog-genomics.org/plink/2.0/) settings.

### Heritability and genetic correlation

For the estimation of the SNP-heritability, two distinct tools were used. With LD score regression (LDSC), it was possible to perform the calculations with the full number of samples as the input for this analysis is the summary statistics. Besides heritability estimates, genetic correlations were also calculated for Aβ42, pTau, tau (to test the similarity in genetic background to pTau), and two previously published AD summary statistics [33, 43]. Precalculated LD scores from the 1,000 Genomes European reference population were obtained online. All estimates were based on HapMap3 SNPs only to ensure high-quality LD score calculations (https://alkesgroup.broadinstitute.org/LDSCORE/). As a rule of thumb, LD Score regression tends to yield very noisy results when applied to datasets with fewer than 5000 individuals (https://github.com/bulik/ldsc/wiki/FAQ) [9]. The summary statistics for the stratified analyses were, therefore, not considered.

For comparison to SNP-heritability estimates of previous studies for Aβ42 and pTau, GCTA v1.9 [74] was applied to the individual-level genotype data of the largest dataset (Netherlands). Other datasets were not considered as the sample size was too low for small standard errors, thereby impossible to draw any meaningful conclusions from the estimates. The restricted maximum likelihood (REML) analysis was performed for the log10-transformed normalized CSF Aβ42 and pTau adjusted for gender, age, and the first 10 principal components. Variance explained could not be calculated for significant loci only as p-values from GWAS results of a large independent sample are unavailable, and calculation in the Dutch sample would be hampered by winners-curse, causing inflation.

### Single-marker association

Genome-wide association analysis for each cohort was performed in PLINK v2.0 [10], using linear regression for the continuous phenotypes Aβ42, tau and pTau. Association tests were adjusted for gender, age, assay type (if applicable), and ten ancestry principal components. Only variants with a minor allele frequency threshold above 0.01 were tested. For smaller cohorts (*n* < 250 individuals) this threshold was set to 0.05 to avoid false positive findings.

Association analyses were repeated for subgroups, stratified according to *APOE*4 status (based on the high-quality (*R*^2^ > 0.8) imputed variants rs429358 and rs7412) or dichotomous Aβ42 status, resulting in the following groups: (1) *APOE*4 (hetero- and homozygous) carriers; (2) *APOE*4 non-carriers; (3) individuals with abnormal Aβ42 levels; and (4) individuals with normal Aβ42 levels. After stratification, cohorts with a minimal sample size of 100 individuals were included. Covariates were those described for the main analyses above.

### Independent replication

A total of 5042 samples from 15 cohorts were included for the replication analysis. The genetic data for NorCog were generated with 2 different genotyping assays. Extensive QC procedures which are detailed elsewhere [6], allowed for joined genetic analyses of these sub-datasets. Variant association testing was performed according to the association analysis section above. For all other replication cohorts, QC, imputation and association testing procedures are described elsewhere [17]. In short, individual and variant QC standards were met, and imputation was performed using the 1000 Genomes Project Phase 3 reference panel. Each dataset was QCed and imputed independently. The additive linear regression model in PLINK v1.9 [10] was used for single-variant analyses.

### Meta-analyses

METAL [71] was used for meta-analyses in stages 1–3 of the per-cohort association results, applying the default approach that utilizes *P* value and direction of effect, while weighted according to sample size. For stage 1, we used the genome-wide threshold for significance of *P* < 5 × 10^−8^, and a suggestive threshold of *P* < 1 × 10^−5^ to select variants to study in Stage 2. Stage 2 variants were considered a replication with *P* < 0.05 and same direction of effect in comparison to stage 1. The genome-wide threshold for significance of *P* < 5 × 10^−8^ was used to defined GWAS hits in stage 3.

### Colocalization

All variants within 1.5 megabases (Mb) of the lead variant of each genomic risk loci were used in the colocalization analysis. The stage 1 GWAS summary statistics were used for the CSF loci aiming consist sample sizes across the variants. Colocalization comparisons were performed to eQTL data for brain and immune-related tissues and cell-types, which were obtained from the eQTL catalog [39] release 5. The microglia data were obtained from Young et al. [77]. GWAS summary statistics for loci comparison to other GWAS studies were obtained from Kunkle et al. [43] for AD, and from Vojinovic et al. [65] for brain ventricular volume. The GWAS data and eQTL data were trimmed so that all variants overlap. Colocalization was performed with the Coloc R package [24], using the coloc.abf function for the approach assuming a single causal variant, and the runsusie and coloc.susie functions to test for colocalization relaxing this assumption to multiple variants. The latter approach was performed for the comparisons to AD and brain volume loci, and LD matrices were calculated with our own individual-level data for the significant CSF loci using LDstore R package [7]. Default priors were used for prior probability of association with the GWAS data and eQTL data. The prior probability of colocalization was set as 5 × 10^−6^ as recommended [67]. Nominal P, sample size and MAF were used when beta and variance of beta were not available for the GWAS data or eQTL data. Colocalizations with a posterior probability > 0.8 were considered successful colocalizations. Comparisons were visualized with the R package LocusCompareR (https://github.com/boxiangliu/locuscomparer).

### Gene-based analysis

Gene-based and gene-set association tests were performed using MAGMA v1.08 [14], which was implemented by FUMA [69]. The per variant association summary statistics for the main results served as the input, where variants were selected if mapped within 18,870 protein-coding genes (with unique ensembl ID). The mean SNP-wise model was implemented. The Bonferroni-corrected significance threshold was set to *P* < 2.65 × 10^–6^, based on the number of tested genes.

### Gene mapping

The genome-wide significant loci of the main results were further explored for promising causal AD genes using FUMA [69], after lifting over the results with genomic build GRCh38 to GRCh37 with the UCSC LiftOver Program (https://genome-store.ucsc.edu/). Two gene mapping strategies were used:

- Positional mapping maps SNPs to genes based on physical distance (within a 10-kb window) from known protein-coding genes in the human reference assembly (GRCh37/hg19).
- eQTL mapping maps SNPs to genes with which they show a significant eQTL association (that is, allelic variation at the SNP is associated with the expression level of that gene). eQTL mapping uses information from 85 brain- and immune-related tissue types in 11 data repositories (BIOSQTL, BloodeQTL, BRAINEAC, CMC, DICE, eQTLcatalogue, eQTLGen, GTEx, PsychENCODE, scRNA_eQTLs, xQTLServer), and is based on cis-eQTLs which can map SNPs to genes up to 1 Mb apart. We used a false discovery rate of 0.05 to define significant eQTL associations.

### Phenome-wide association studies (PheWAS)

We conducted phenome-wide association studies (PheWAS) on the top SNPs, rs4844610, rs429358, rs744373, rs9877502, rs4843559. A PheWAS starts out with a single to a few variants of interest that are systematically being tested for association to many phenotypes. We used the ‘phewas’ function of the R-package ‘ieugwasr’ [19, 31]. Using this function, we searched traits that associate with the list of SNPs with *P* < 1 × 10^–7^ in all GWAS harmonized summary statistics in the MRC IEU OpenGWAS data infrastructure [31]. In short, this enables us to screen for other traits to which these SNPs are associated. The database (May 2021) includes the GWAS summary statistics of 19,649 traits.

### Association with CSF proteomics

We associated the lead variants near *GMNC* (rs9877502) and in *C16orf95* (rs4843559) with CSF proteomics data of two different sources (EMIF-AD MBD and Knight-ADRC). For the EMIF-AD MBD data, a total of 2,136 proteins were quantified centrally using 11-plex tandem mass tag spectrometry in 366 individuals from the EMIF-AD MBD study [61] (subset of Amsterdam Dementia Cohort within EADB). We selected proteins with a maximum of 50% missing values. For related proteins that had identical values due to fragment aspecificity, we randomly selected one protein for analysis (52 proteins were excluded). Out of the 2136 proteins quantified, 1282 (55.4%) proteins respected these criteria and were included in the study.

For the Knight-ADRC data, levels of 1305 proteins were quantified using the SOMAscan assay, a multiplexed, aptamer-based platform CSF (*n* = 717) [73]. Quality control was performed at the sample and aptamer levels using control aptamers (positive and negative controls) and calibrator samples. As described in detail [73], additional quality control was performed that included limit of detection cut-off, scale factor, coefficient of variation, and outlier variation. Only proteins with a call rate higher than 85% call rate were included. A total of 713 proteins passed quality control. pQTL analyses was performed and reported in previous studies [73].

g:Profiler and Enrichment map [44], a Cytoscape App, were used to perform pathway enrichment analyses on proteins with a certain level of association (EMIF-AD MBD: *P* < 0.05; Knight-ADRC: *P* < 0.004, corresponding to proteins with a similar effect size as in EMIF-AD MBD). The results are shown as functionally grouped networks. We used GO biological processes and Reactome as ontology sources. For this explorative analysis, only pathways with *P* < 0.05 (corrected for multiple testing) are shown.

### Effects of AD-associated variants on Aβ42 and pTau

We assessed the most recent GWAS [6] for AD and extracted the top loci of 83 variants (excluding *APOE* ɛ4 and *APOE* ɛ2) that showed genome-wide significant association with AD [6]. We extracted Z-scores and *P* values and plotted them in a heatmap. Rows and columns were clustered using Euclidean distances and average hierarchical clustering. We performed a gene-set enrichment analysis to find molecular pathways enriched within each cluster. The SNP-gene assignment corresponds to the one described in the recent main EADB GWAS [6], including several annotation strategies. When multiple genes were reported to associate with the same SNP (rs12590654 near *SLC24A4*/*RIN3*, rs7225151 near *SCIMP*/*RABEP1*, rs6846529 near *CLNK*/*HS3ST1*, rs7384878 near *ZCWPW1*/*NYAP1* and rs10437655 near *CELF1*/*SPI1*), we considered both genes for the gene-set enrichment analysis. In addition, for SNP rs6605556, located in the complex HLA region, we considered HLA-DRB1 gene (eQTL in blood with rs6605556), and for SNPs rs7157106 and rs10131280, both located in the gene-dense IGH region, we considered IGHG2 and IGHV2-70 (eQTLs in blood with rs7157106 and rs10131280, respectively). The gene-set enrichment analysis was performed specifying Biological Processes from Gene Ontology [1, 5] as gene-set and correcting *P* values with Bonferroni. Biological pathways were considered significant at corrected *P* < 0.01. To help with the interpretation of each cluster’s function, we plot the most recurring words of the significant terms underlying each cluster using wordclouds. The following R packages were used for these analysis: gprofiler2 [41] and wordcloud2 (https://github.com/lchiffon/wordcloud2).

## Results

The overview of the study design is illustrated in Online Resource 2—Fig. 1. The GWA results from 16 studies were combined in stage 1. Variants that reached a suggestive level of significance (*P* < 1 × 10^–5^) were subsequently evaluated in an independent sample from 15 studies in stage 2. Finally, the results of stage 1 and stage 2 analyses were combined in stage 3. Detailed information on study participants, CSF acquisition and genotyping is provided in Table 1 and Online Resource 1—Tables 1 and 2. The results for tau and pTau are strongly correlated (*r*_g_ = 0.94; *P* = 1.86 × 10^–118^), and therefore only pTau findings are reported.

### Genetic architecture

The fraction of variance in Aβ42 and pTau protein levels that could be explained by the additive effect of the genetic variants tested, was estimated on 0.13 (SE = 0.06) and 0.21 (SE = 0.07) by LDSC, respectively. These SNP-heritabilities are substantially higher than the 0.07 previously estimated with LDSC for the diagnosis AD [80], or similarly reported for AD by this study using the same LDSC method on more recent public GWAS summary statistics of AD [33, 43] (Online Resource 1—Table 3). GCTA estimated the SNP-heritability to be higher for both Aβ42 and pTau, namely 0.27 (SE = 0.13) and 0.34 (SE = 0.12), respectively. Both methods are reporting a higher SNP-heritability for pTau than for Aβ42. Genetic correlation estimates with AD GWAS summary statistics are described in Online Resource 1—Results and Online Resource 1—Table 4.

### GWAS variants associated with CSF Aβ42 and pTau

The stage 1 meta-analyses (QQ plots and lambda shown in Online Resource 2—Fig. 14) identified 4 independent significant variant associations, 1 for Aβ42 and 3 for pTau (Table 2). The strongest associations were observed for both Aβ42 and pTau in the *APOE* locus (Table 2). The variant that determines the *APOE* ɛ4 allele (rs429358-C) decreased Aβ42 (*Z* = − 36.29; *P* = 2.0 × 10^–288^) and increased pTau (*Z* = 18.31; *P* = 6.87 × 10^–75^) in CSF. In contrast, the variant determining the *APOE* ɛ2 haplotype (rs7412-T) increased Aβ42 (*Z* = 11.97; *P* = 5.09 × 10^–33^) and decreased pTau (*Z* = − 6.59; *P* = 4.49 × 10^–11^). The *APOE* ɛ2 association was replicated in stage 2 (Aβ42: *Z* = 7.27; *P* = 3.73 × 10^–13^, and pTau: *Z* = − 6.43; *P* = 1.26 × 10^–10^), and *APOE* ɛ4 with rs4420638-G for Aβ42 (*Z* = − 25.51; *P* = 1.57 × 10^–143^), and with rs769449-A for pTau (*Z* = 13.83; *P* = 1.66 × 10^–43^), both variants in high linkage disequilibrium with rs429358, as the original *APOE* ɛ4 variant was not genotyped or imputed in the replication datasets.

**Table 2:** Meta-analysis association results for the 3 stages

Protein	Locus	rsID	Chr	Pos (hg38)	A1	A2	Stage 1				Stage 2			Stage 3		
							Freq A1	*n*	*Z*	*P*	*n*	*Z*	*P*	*n*	*Z*	*P*
AB42	*1q32.2 (CR1)*	rs4844610	1	207,629,207	A	C	0.20	8074	− 4.87	1.13E–06	5015	− 3.54	4.08E–04	13,089	− 6.01	1.84E–09
	*APOE*	rs429358	19	44,908,684	C	T	0.29	8074	− 36.29	2.00E–288	4488	− 25.51	1.57E–143	12,562	− 41.68	1.00E–379
pTau	*2q14.3 (BIN1)*	rs744373	2	127,137,039	G	A	0.30	7798	5.39	6.99E–08	3948	3.03	2.48E–03	11,746	6.15	7.88E–10
	*3q28 (GMNC)*	rs9877502	3	190,951,729	A	G	0.39	7798	9.06	1.28E–19	4742	8.97	3.00E–19	12,540	12.66	9.65E–37
	*16q24.2 (C16orf95)*	rs4843559	16	87,191,825	G	A	0.38	7798	6.41	1.49E–10	3985	3.88	1.03E–04	11,783	7.47	8.03E–14
	*APOE*	rs429358	19	44,908,684	C	T	0.28	7798	18.31	6.87E–75	4741	13.83	1.66E–43	12,539	20.65	9.59E–95

Z-statistic summarizes the magnitude and the direction of effect relative to the effect allele. For APOE stage 2 and stage 3, the statistics are reported for the most significant stage 3 variants rs4420638 (G allele) for AB42 and rs769449 (A allele) for pTau, as rs429358 was not genotyped or imputed in the replication datasets

*rsID* ID number in SNP database, *Chr* chromosome, *Pos* genomic base pair position, *A1* effect allele, *A2* other allele, *Freq A1* allele frequency effect allele, *n* sample size, *P* *p* value

In stage 1, no other significant loci were observed for Aβ42. For pTau, we further identified significant associations mapping to two chromosomal regions at 3q28 and 16q24.2 (Table 2). The 3q28-locus (*Z* = 9.06; *P* = 1.28 × 10^–19^) also known as the *GMNC* locus, was reported previously for its association with pTau [17]. The cohorts from this previous study are the replication cohorts of the current study, thereby logically *GMNC* was replicated (*Z* = 8.97; *P* = 3.00 × 10^–19^). The 16q24.2 locus (*Z* = 6.41; *P* = 1.49 × 10^–10^), which is novel for pTau, was replicated in the stage 2 meta-analysis (*Z* = 3.88; *P* = 1.03 × 10^–04^).

Subsequently, the results from all individual studies were combined in the stage 3 meta-analysis (*N* = 13,116). In stage 3, two well-known AD loci showed additional genome-wide significant associations (Fig. 1, Table 2) with Aβ42 in chromosomal region 1q32.2 (*Z* = − 6.01; *P* = 1.84 × 10^–9^, *CR1;* Fig. 1a), and with pTau for the region 2q14.3 (*Z* = 6.15; *P* = 7.88 × 10^–10^, *BIN1;* Fig. 1b). The per-cohort and zoomed in genomic location details of all significantly associated loci of stage 3 are visualized in Online Resource 2—Figs. 15–20. Colocalization analyses (results detailed in Online Resource 1—Table 5; Online Resource 2—Fig. 21 and 22) showed colocalization of the *CR1* (posterior probability = 0.97) and *BIN1* (posterior probability = 0.82) loci to these loci in a recent AD GWAS [43]. For the *BIN1* locus of the AD GWAS two independent causal signals were observed, of which our *BIN1* locus colocalized (posterior probability = 0.84) with the first signal that was tagged by rs6733839, which is the most significant variant of the *BIN1* locus of the AD GWAS.

Explorative meta-analyses were repeated stratified for *APOE* (*APOE* ɛ4 carriers (*n* = 3240) vs. *APOE* ɛ4 non-carriers (*n* = 3201)) and amyloid status (Amyloid normal levels (*n* = 3182) vs. amyloid abnormal levels (*n* = 3775)) for stage 1 (QQ plots and lambda shown in Online Resource 2—Figs. 23 and 24), of which the results are visualized in Online Resource 2—Figs. 25 and 26, and detailed in Online Resource 2—Results and Online Resource 1—Table 6. Besides the *APOE* and *GMNC* loci, two novel loci are observed that have previously not been linked to any AD phenotype.

### Functional interpretation

To interpret the functional effects of the identified variants beyond AD, we performed gene prioritization (based on positional mapping, gene-based association results, and brain and immune eQTL annotations) using FUMA [69], colocalization analyses and PheWAS. The results of the FUMA annotation are detailed in the Online Resource 2—Results and Online Resource 1—Table 7. Ten of our CSF Aβ42 and pTau loci colocalized with one of the brain or immune eQTLs from the 63 tested datasets, which are reported in Online Resource 1—Table 8. The *APOE* locus for both Aβ42 and pTau colocalized with an eQTL for *NKPD1* in a specific immune helper T cell. The *CR1* locus for AB42 colocalized with an eQTL for the *CR1* gene in 6 brain tissues (hippocampus, caudate, putamen, dorsolateral prefrontal cortex, frontal cortex, cortex). Additionally for the dorsolateral prefrontal cortex, the CR1 locus also colocalized with an eQTL for the *AL137789.1* gene. The *BIN1* locus colocalized with an eQTL for *BIN1* in lymphoblastoid cell lines.

For the PheWAS, using data from publicly available genome-wide association studies (*N* = 19,649) of the five top variants yielded 529 associations at *P* < 1 × 10^–7^ (Online Resource 1—Table 9). The majority is the known wide range of 490 traits associations with the APOE ɛ4 allele. For the other variants 39 associations were reported for 27 unique traits. These traits can be categorized in three groups: traits related to brain ventricular volumes in particular the lateral-ventricle (*GMNC* and *C16orf95*), Alzheimer’s disease diagnosis (*BIN1* and *CR1*), and measures of blood cell/lymphocyte counts (*CR1*). The regional pTau associations of *GMNC* and *C16orf95* overlapped with ventricular volume (Fig. 2). Colocalization results (Online Resource 1—Table 5; Online Resource 2—Figs. 27 and 28) imply the same causal variant for *C16orf95* (posterior probability = 0.93). The *GMNC* locus cannot be convincingly explained by the same causal variant (posterior probability = 0.63). The coloc method combined with Susie reports only 1 causal signal for both GWAS. Of note, the colocalization probability improves to 0.74 when first reducing the *GMNC-*locus variants to a credible set of variants. Although these results are subthreshold to the predefined posterior probability limit of 0.8, they do imply that the same causal variant is likely underlying the *GMNC* association signals for pTau and AD.

Because of the overlap in effect of *GMNC* and *C16orf95,* we hypothesized that these two loci affect the same biological pathways. We explored this hypothesis using CSF proteomics datasets of the EMIF consortium with 1284 quantified proteins and of Knight-ADRC with 696 quantified proteins (of which 42% overlap with the EMIF-AD MBD proteins). For *GMNC* there were 279 (22%) proteins associated in the EMIF-AD MBD data (Online Resource 1—Table 10) and 255 (36%) proteins in the Knight-ADRC data (Online Resource 1—Table 11). *C16orf95* could only be tested in the EMIF-AD MBD data in which 73 (6%) proteins were associated (Online Resource 1—Table 10). Only 2 proteins (CDH9 and DPP6) overlapped between the 2 loci. We studied the overlap in affected pathways between the associated protein lists. For *GMNC*, consistent functional group networks between the 2 tested datasets were axon guidance and ephrin signaling (Online Resource 2—Fig. 29, Online Resource 1—Tables 12 and 13), while for *C16orf95* (only based on the EMIF-AD MBD data) glycosaminoglycan metabolism and ECM organization were overrepresented functional groups (Online Resource 2—Fig. 30, Online Resource 1—Table 14). There was little overlap between the loci in the pathways that emerged from the protein lists.

### Relation to AD-associated genetic variants

Because of the evident overlap in etiology with clinical AD dementia, we examined the association of all known AD loci (excluding the APOE locus) with CSF Aβ42 and pTau. This analysis has an explorative character as the small contribution of each individual AD loci to CSF amyloid and pTau is in general reflected in moderate effect sizes and association signals. However, patterns of reasonable signal could facilitate the generation of biological hypotheses to test in future experiments. The results are shown in the heatmap of Fig. 3 and Online Resource 1—Tables 15 and 16. The variants could be clustered in 4 groups of AD-associated genes based on their associations with Aβ42 and pTau. The first cluster of 14 variants showed strong association with both decreased levels of Aβ42 and increased levels of pTau in CSF. A pathway enrichment analysis of the genes associated with the variants showed 29 GO terms enriched and ‘amyloid’ is the common denominator in the names of these terms, of which the signal is mostly driven by *BIN1, PICALM ABCA7* and *CLU*. The second cluster contained 21 variants and included genes that have also been related to other dementia types (e.g., *GRN, TMEM107B, SNX1, MAPT, CTSB* and *CTSH*). This cluster was associated with decreased pTau levels, an no general effect on Aβ42 levels. Pathway analysis of the genes suggests an enrichment for 8 GO terms of which the names have ‘immune’ as a common denominator that is mostly driven by 12 genes of which *TREM2* and *GRN* are the most frequent contributors. The third cluster consisted of 22 variants, which were related to decreased levels of Aβ42 but not increased levels of pTau. Nine GO terms were enriched and ‘migration’ and ‘tyrosine’ are the words that occur most often in these terms, though each word only based on 2 GO terms each. The last cluster of 20 variants group because they increased pTau, but did not decrease Aβ42 levels. No GO terms are significantly enriched in this gene cluster.

## Discussion

We identified 2 loci (*CR1* and *APOE*) for Aβ42, and 4 loci (*BIN1, GMNC, C16orf95* and *APOE*) for pTau in a total of 13,116 individuals (discovery *n* = 8074; replication *n* = 5402 individuals). In concordance with previous GWAS studies [12, 17], both proteins showed the strongest association for *APOE*, where *APOE ɛ*4 decreased amyloid-beta levels and increased pTau levels, while *APOE* ɛ2 had the opposite effect. We confirmed *GMNC* as a risk factor for CSF pTau levels. We identified *CR1* as a novel locus for CSF Aβ42 levels, and we observed 2 novel loci (*BIN1* and *C16orf95*) for CSF pTau levels. So other than *APOE,* no risk loci overlap is observed for Aβ42 and pTau, implying at least partly separate genetic backgrounds for both pathological hallmarks. Amyloid-beta appears to be dominated by the effect of *APOE*, while pTau is influenced by multiple genetic components. Such a divergence in genetic influences is not in concordance with a genetic etiology where accumulation of Tau tangles is a direct downstream effect of amyloid plaque formation, as proposed by the amyloid cascade theory [29, 30]. Rather, it seems that these pathologies have an independent component in its origin, as already extensively reviewed [64], thereby implying a dual-cascade hypothesis in which sporadic AD pathogenesis is caused by defects in correlated but independent cellular processes. This is highly relevant biological knowledge for the development of potential AD treatments. The limited clinical efficacy of agents that aim to reduce beta-amyloid plaques might potentially be due to this [76].

In line with this implication is the observed difference in genetic subgroups based on CSF protein patterns as proposed by the explorative cluster analysis, suggesting multiple Aβ42 and pTau related biological pathways to be involved in the etiology AD. The first genetic subgroup is defined by the word ‘amyloid’, where *BIN1, PICALM, ABCA7* and *CLU* contribute most to this signal. The involvement of these genes in amyloid-beta pathology is supported by previous studies [15, 46, 63, 79]. ‘Immune’ is labeling the second subgroup, thereby implying that the genes included in this cluster are influencing AD by altering immune responses. The largest genetic drivers of this signal are *TREM2* and *GRN* which have been previously described for their functional role in AD via the immune system [45, 54]. A substantial involvement of the immune system in AD is a generally accepted concept, supported by clinical, functional and genetic research [8]. As mentioned in the Results section, this second cluster includes genes that have also been related to other dementia types (e.g., *GRN, TMEM107B, SNX1, MAPT, CTSB* and *CTSH*). This is in line with more recent insights where dysfunction of the immune response is seen as a common cause for multiple neurodegenerative diseases [28]. The third and fourth clusters are less straightforward to interpret as many terms are similarly enriched, or no significant enrichment is observed. The definition of the first 3 subclasses in its current state is the best approximate within reach, though presumably not a perfect reflection of the genuine genetic etiology of AD. Subsequent genetic studies with larger sample sizes are anticipated to facilitate an improved understanding of the biological implications of the underlying genetic subclasses.

The variety in subclasses of genetic contributors for AD etiology could mean that different patient groups might benefit from distinct AD treatment depending on the biological pathway that is affected. Although, our genetic results in its current state are not applicable for clinical trials, we believe by improving the definition of the amyloid-tau clusters (e.g., by increasing sample sizes), it will be possible to assign individuals to one of the groups based on the distribution of their AD risk variants. For example, individuals with relatively more AD risk variants in the first cluster should preferably be included in a clinical trial addressing amyloid formation, while individuals with higher sub PRS scores for the second immune cluster, would have a higher probability benefitting from a clinical trial targeting the immune system. Alternatively, when sample sizes are increasing for future CSF amyloid and tau GWAS, an amyloid or tau PRS could be calculated to identify individuals at high genetic risk for amyloid and tau, thereby defining the individuals that should be included in trials aiming to reduce amyloid plagues or tau neurofibrillary tangles, respectively.

As mentioned above, *APOE* is the strongest locus for both CSF Aβ42 and pTau. The functional implications of the relation between *APOE* and the 2 pathological hallmarks has been excessively studied and reviewed in the scientific field of Alzheimer’s disease [20, 32, 60, 64]. In short, studies in human and mice models have shown higher Aβ42 levels and plaque load for APOE4 carriers. Whether this is a result of gain of toxic effect (e.g., enhanced ability of the APOE4 isoform to bind to Aβ42 [40]), or loss of defensive mechanism (e.g., less effective microglial response[20]), or a combination of both, remains to be elucidated. For Tau, studies have shown hyperphosphorylation and faster accumulation for APOE4 carriers, both in mice and human models [32, 64]. Again, the functional route through which APOE is affecting the Tau aggregates is unknown, and multiple cellular processes are being proposed as mediators, including neuronal endocytosis, lipid metabolism and glial function. An important functional observation though is the influence of APOE4 on tau accumulation in absence of Aβ42 pathology [55, 68], which is in agreement with the genetic implications of this current study. An unexpected observation for the APOE locus in this study is the successful colocalization with an eQTL for the *NKPD1* in a specific type of T cells. However, due to the convincing functional involvement of *APOE* in AD pathogenesis via the presence of cysteine or arginine at APOE amino acid residuals 112 and 158, that we anticipate this successful *NKPD1* eQTL colocalization result to be a false positive.

The *CR1-*locus findings are in agreement with the well-known observed association for AD risk, where rs6656401 (*R*^2^ = 0.88 with lead SNP of current study) carriers are more susceptible for AD. The similarity in these association signals is strengthened by the convincing colocalization of the *CR1* locus between our CSF pTau observation and the *CR1* locus of Kunkle et al. [43]. We furthermore observe a successful colocalization with an eQTL for the CR1 gene in multiple brain regions. In concordance with this observation is functional work on the effect of CR1 further which suggests that CR1 is involved in AD pathogenesis by regulating Aβ42 clearance in the brain itself, but also peripherally in blood cells [81]. More recent research in red blood cells of AD patients showed deficient CR1 immunoreactivity, including CR1-mediated capture of circulating amyloid-beta [35]. They observed decreased CR1 protein levels in red blood cells for *CR1* SNPs that associate with higher AD risk. The second novel locus in this study is *BIN1* for CSF pTau, for which we observe a successful colocalization for an eTQL for the *BIN1* gene in lymphoblastoid cell lines, implying *BIN1* to be the causal gene for this locus. The *BIN1-*locus furthermore localizes with the same locus in the AD GWAS, implying that a causal variant of the *BIN1-*locus for AD contributes to AD pathogenesis via tau pathology. *BIN1* has already been linked to Tau pathology in several functional studies, first shown in fruit flies where a decrease in the *BIN1* ortholog gene expression suppressed Tau-mediated neurotoxicity [11]. More recently, research in mice showed physical protein interaction between BIN1 and Tau [51], and BIN1 involvement in Tau-dependent hyperexcitability in AD [66]. In human subjects, *BIN1-*carriers were associated with lower memory performance, mediated by higher tau-PET levels [22]. Our observed *BIN1-*Tau association contributes valuable knowledge on this topic, by observing the same trend in a substantial larger study (89 vs 13,118 individuals) using different techniques to measure Tau pathology (PET vs. CSF). We provide in vivo confirmation that *CR1* is associated with AD via Aβ42, while *BIN1* relates to Tau pathology.

The third novel locus in this study is the region on genomic location 16q24.2 for pTau, of which the strongest associated variants are located within intronic regions of *C16orf95*. This locus has not been linked to (p)Tau pathology or AD in previous research, though it associated to lateral ventricular volume in the CHARGE study, including 23.5 k healthy individuals [65]. Similarly, the 3q28-locus for pTau from our findings colocalizes inconclusively (posterior probability of 0.73) to lateral ventricular volume by the CHARGE study, implying that the same genetic risk factors contribute to both phenotypes, strengthening the notion that neurodegeneration and (p)Tau pathology are highly correlated. 3q28 has been linked to (p)Tau by previous CSF studies in dementia cohorts and was identified as the *GMNC* locus [12, 17]. In comparison to the latest GWAS of Deming et al. [17], increasing the sample size with a small 10 k individuals in this study strengthens the association of this locus from 3.07 × 10^–11^ to 1.19 × 10^–36^, thereby turning it into a well-established locus for pTau pathology (similar for Tau: Deming *P* = 3.07 × 10^−11^ (*n* = 3146); current *P* = 9.65 × 10^–37^ (*n* = 12,540). The *C16orf95*-locus convincingly colocalized with the same locus in ventricular volume. The formerly reported directions of effect of *GMNC* and *C16orf95* for ventricular volume are counterintuitive. For both loci the allele that associated with an increase in pTau pathology in our dementia cohorts associated with a smaller ventricular volume, implying less neurodegeneration. We explored if these loci work through the same biological pathways using CSF proteomics data. The consistently highlighted functional groups for the *GMNC* locus (axon guidance and ephrin signaling) were different than for the *C16orf95-*locus (extracellular matrix components), thereby implying at least partly distinct functional routes via which they influence pTau protein levels in CSF. The power of the proteomics dataset is rather limited. We anticipate that these analyses would benefit and potentially find overlapping biological pathways, when AD proteomics datasets at hand will increase in sample size, or apply the same proteomics assay (rather than using different ones with little overlap in proteins).

We furthermore identified two novel loci in the stratified analyses for Aβ42 levels: 7q11.22 for *APOE* ɛ4 non-carriers and 12q13.3 in individuals with abnormal amyloid levels. We were unable to test for replication of these loci in independent replication datasets, as such analyses were not performed. Neither loci have been previously linked to AD, or any other trait. The lead SNP for the locus on chromosome 7 is a common intronic variant for the lncRNA *LOC105375341*, which according to GTEx is only expressed in testis and prostate, and thereby not a promising causal gene for AD or AD-related phenotypes. The locus on chromosome 12 consists of just one rare intronic variant that was only detected in the Dutch cohort with the largest sample size (*n* = 498). Future studies including more cohorts of large sample sizes are required to study this rare variant in more detail.

Besides *APOE* and *GMNC*, no other loci from the latest GWAS of CSF amyloid and tau [17] were replicated by this study. For Aβ42, it was not possible to test the *GLIS1*-locus as this variant was not observed in our data, which is in concordance with the gnomAD browser [37] reporting extremely low coverage and a MAF of 0.007 for rs185031519, the strongest *GLIS1* SNP in Deming et al. For the other loci that we were unable to replicate (*SERPINB1* for Aβ42; and *GLIS3*, *PCDH8*, *CTDP1* for pTau), there were no significant associations (*P* > 0.05) despite substantial sample sizes (*n* > 7000). These differences in findings might be due to differences in study design, for example inclusion of cohorts with other diagnoses and/or differences in analysis strategies.

The GCTA-based SNP-heritability estimates of 27% and 34% for Aβ42 and pTau, respectively, are in a similar range to the estimates calculated by Deming et al. [17] (36% for Aβ42 and 25% for pTau). Notable is that the previous study estimated Aβ42 to be most heritable, while we observed the highest estimate for pTau, though the standard errors were about 10% for both studies. Our LDSC-based SNP-heritabilities show a similar trend with higher estimates for pTau. Furthermore, these LDSC-based 13% for Aβ42 and 21% for pTau protein levels are considerably higher than the 7% previously observed for the diagnosis AD. The higher heritability for the tested CSF protein levels strengthens the assumption that more objective measurable biological properties are more strongly associated with AD pathogenesis than the diagnostic classifications.

Due to this assumption, the number of risk loci identified in this study might be lower than anticipated. However, our study design is suboptimal for obtaining the most powerful model for genetic association identification. Meta-analyzing 31 heterogeneous cohorts (differences in genotyping array, imputation references, CSF protein assays, and patient/control ratios) is more challenging rather than analyzing a homogeneous dataset of similar sample size. Alternatively, increasing the sample size by adding more cohorts to increase power is presumably a more feasible approach for the future.

In conclusion, the current findings clearly show that studying the genetic effects of AD-related endophenotypes has the potential to reveal novel associations, and highlight important biological insights. The clear distinction in genetic findings for amyloid-beta and tau emphasizes the (partly) genetic independence of these two biological mechanisms in AD pathogenesis. Moreover, the identification of *CR1* and *BIN1*, which are the second and third strongest associated AD locus after *APOE*, furthermore implies that by increasing sample size of genetic analysis in CSF biomarkers it will become more apparent through which biological mechanisms certain AD loci have their effect on AD pathogenesis. Even larger collaborative efforts with more homogeneous sample definitions are, therefore, encouraged to be undertaken to enhance our genetic understanding of AD, ultimately leading to improved biological knowledge for the development of drug treatment.

## Supplementary Information

Below is the link to the electronic supplementary material.

Supplementary file1 (XLSX 306 kb)

Supplementary file2 (PDF 2115 kb)

## Data Availability

Genome-wide summary statistics have been deposited to the European Bioinformatics Institute GWAS Catalog (https://www.ebi.ac.uk/gwas/) under accession IDs GCST90129599 and GCST90129600.
